# Fatal fat embolism syndrome during posterior spinal fusion surgery: A case report and literature review

**DOI:** 10.1097/MD.0000000000028381

**Published:** 2021-12-23

**Authors:** Tadatsugu Morimoto, Takaomi Kobayashi, Tomohito Yoshihara, Masatsugu Tsukamoto, Keita Kai, Masaaki Mawatari

**Affiliations:** a Department of Orthopaedic Surgery, Faculty of Medicine, Saga University, 5-1-1 Nabeshima, Saga, Japan; b Department of pathology, Faculty of Medicine, Saga University, 5-1-1 Nabeshima, Saga, Japan.

**Keywords:** allograft, bone augmentation, case report, fat embolism syndrome, posterior spinal fusion surgery

## Abstract

**Rationale:**

Fat embolism syndrome (FES) is a rare but potentially lethal complication. Although serious FES is associated with long bone fractures and major joint surgery, the number of patients who develop fatal FES intraoperatively is probably higher than the described number. We herein report an extremely rare autopsy-confirmed case of fatal FES during posterior spinal fusion to enhance pedicle screw (PS) fixation with allograft bone augmentation.

**Patient Concerns:**

A 74-year-old woman came to the hospital complaining of back pain, lower extremity pain and numbness, and intermittent claudication.

**Diagnosis:**

She was diagnosed with lumbar degenerative scoliosis and lumbar spinal canal stenosis based on imaging findings.

**Interventions:**

During posterior spinal fusion to enhance pedicle screw fixation with allograft bone augmentation, her blood pressure and oxygen saturation dropped significantly, so the operation was stopped, and cardiopulmonary resuscitation was performed. Chest computed tomography demonstrated bilateral diffuse alveolar infiltrates.

**Outcomes:**

The patient died three days later due to fat embolism. The autopsy revealed diffuse myocardial ischemia and diffuse alveolar damage. Numerous fat emboli were observed at lung, kidney and spleen and small necrotic bone fragments, possibly derived from allograft bone debris, were found in the peripheral pulmonary artery.

**Lessons:**

Fatal FES associated to seemingly harmless isolated osteoporotic vertebral fractures-vertebroplasty and posterior spinal fusion has been reported. The mechanism was hypothesized to be that both vertebral fractures and spine surgery have the potential to involve bone marrow, thereby increasing intraosseous pressure, and this pressure dislodges fat and bone marrow and pushes them out into the venous circulation, causing systemic inflammation.

This is the first report to show histological evidence that the allografted bone embolized to the lungs. Although several reports have indicated that inserting reinforcing materials into the tapped screw holes can enhance the pedicle screw fixation, this procedure may cause severe FES due to fat and debris of material augmentation (i.e. cement, hydroxyapatite, allograft bone). It is important for physicians, especially spinal surgeons, and anesthetists, to be aware of the potential for FES to occur during spinal surgery, which can cause serious complications in a small minority of patients.

## 1 Introduction

Fat embolism syndrome (FES) is a rare but potentially lethal complication.^[1,2]^ Although most previously reported cases of serious FES have been associated with long bone fractures and major joint surgery,^[1,2]^ the number of patients who develop fatal FES intraoperatively is probably higher than the described number. We herein report an extremely autopsy-confirmed rare case of fatal FES during posterior spinal fusion to enhance pedicle screw (PS) fixation with allograft bone augmentation. To our knowledge, this is the first report to show histological evidence that not only fat but also the allografted bone embolized to the lungs.

## 2 Case presentation

**Figure 1:**
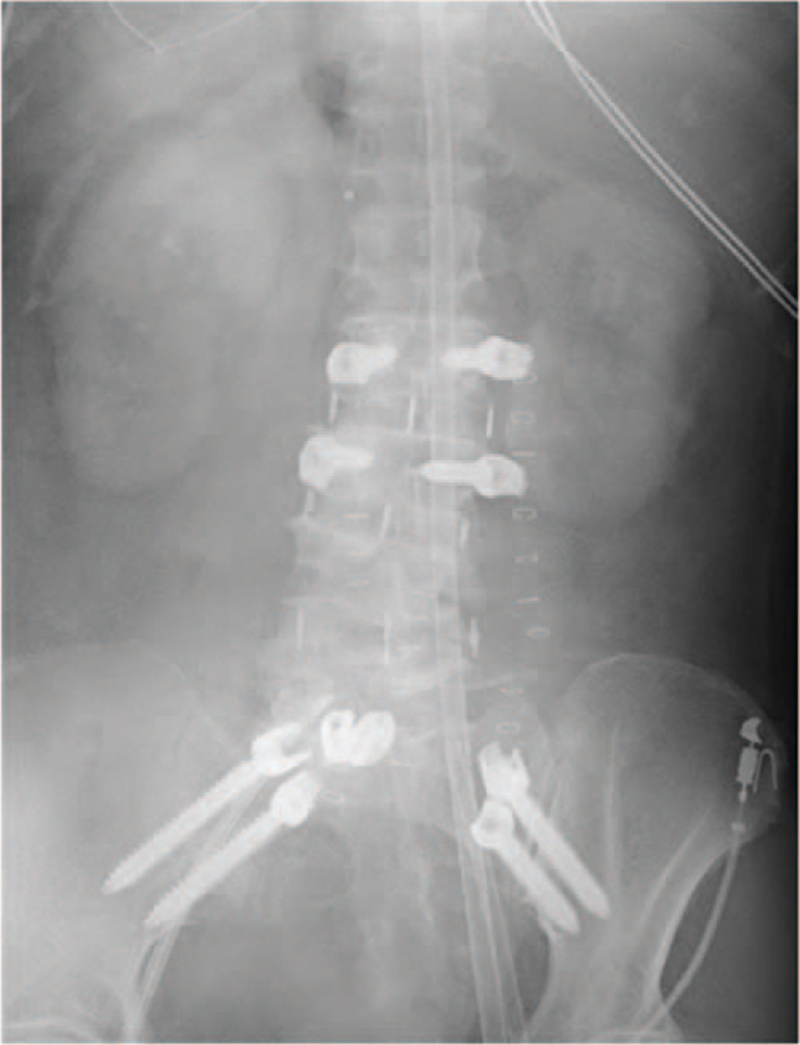
Lumbar X-ray at four days after anterior lumbar interbody fusion. During placement of the L5/S1 interbody fusion cage and iliac screws, followed by the insertion of a pedicle screw with augmented allografted bone, the procedure was interrupted for cardiopulmonary resuscitation due to shock vitals.

**Figure 2:**
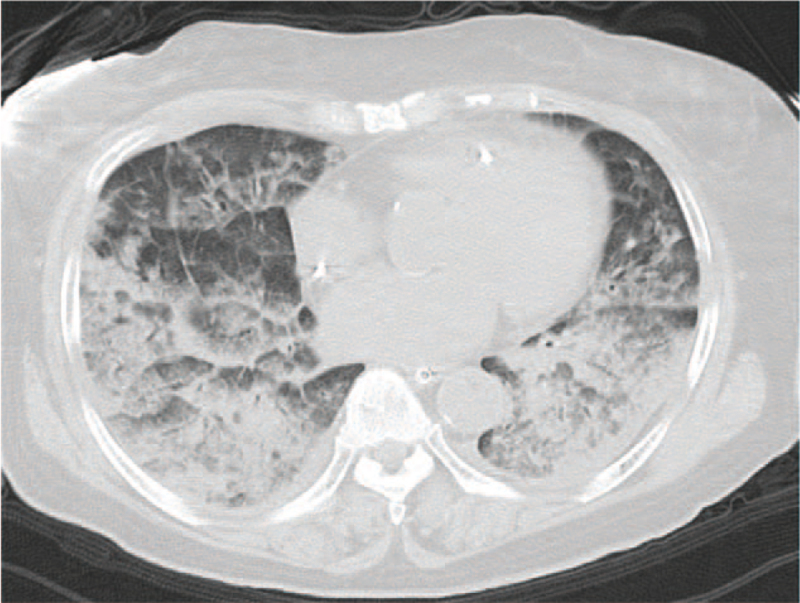
Computed tomography of the lungs. Chest computed tomography showed bilateral diffuse alveolar infiltrates.

A 74-year-old woman with severe back pain and intermittent claudication due to lumbar degenerative scoliosis and spinal canal stenosis. She showed no abnormalities in her physique or on laboratory tests. Retroperitoneal transpsoas approach with lateral vertebral body fusion (L2/3-L4/5) was performed first without any intraoperative or postoperative complications. Four days after the initial operation, posterior spinal fusion (L2-iliac) was planned. L5/S1 posterior interbody fusion with a titanium cage and fixation of four iliac screws were performed, followed by PS fixation. Allograft bone was inserted into the screw holes to increase the PS fixation at L2 and L3 (Fig. 1). When L2 and L3 PS were placed, the systolic blood pressure decreased abruptly, and it became difficult to maintain the systolic blood pressure. Following the use of adrenaline, the invasive arterial pressure improved, but the oxygen saturation fell from 98% to 64%. The patient was promptly returned to the supine position, and cardiopulmonary resuscitation was performed, after which the patient was managed on a ventilator. Chest computed tomography demonstrated bilateral diffuse alveolar infiltrates, suspected FES (Fig. 2). Unfortunately, the patient died three days after entering the intensive-care unit.

**Figure 3:**
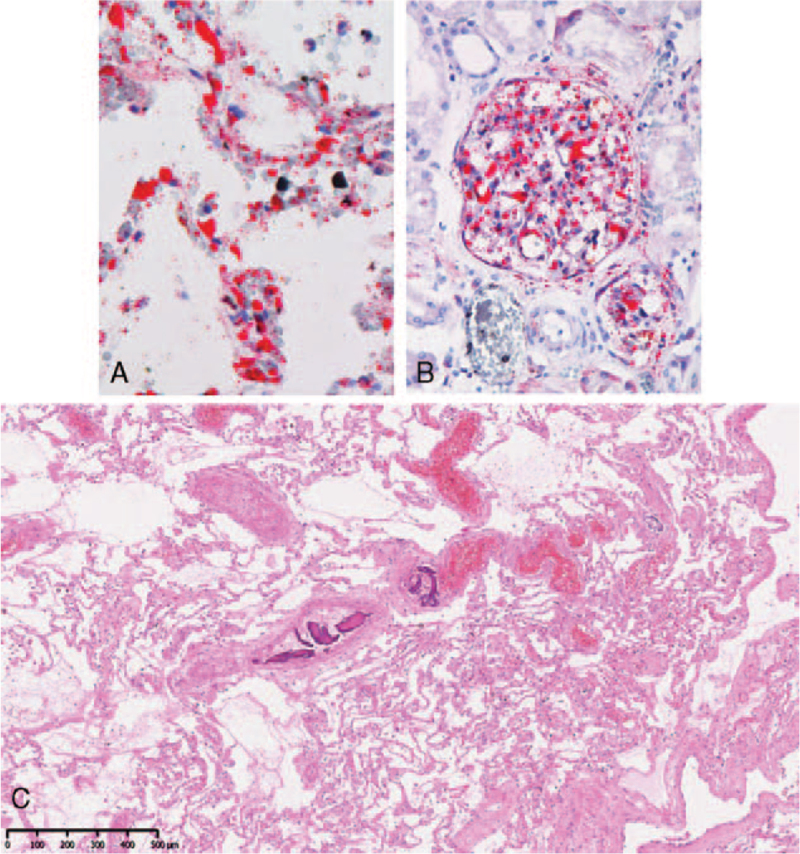
Histological findings. Numerous fat emboli were found at alveolar of the lung (A: Oil-red staining, × 400) and at glomerulus of the kidney (B: Oil-red staining, × 200). (C) Small necrotic bone fragments were found in the peripheral pulmonary artery.

The autopsy revealed diffuse myocardial infarction and severe diffuse alveolar damage. Numerous fat emboli were observed at lung (Fig. 3A), kidney (Fig. 3B) and spleen. Unusually, small necrotic bone fragments, possibly derived from allograft bone debris, were found in the peripheral pulmonary artery (Fig. 3C). The cause of death was determined to be systemic fat embolism.

## 3 Discussion

Fatal FES related to seemingly harmless isolated osteoporotic vertebral fractures,^[3,4]^ vertebroplasty ^[5]^ and posterior spinal fusion ^[6–11]^ has been reported. The mechanism was hypothesized to be that both vertebral fracture and spinal surgery can involve the bone marrow, which increases the intraosseous pressure; this pressure then dislodges the fat and bone marrow, pushing them out into the venous circulation ^[1,5]^ and causing systematic inflammation.^[1,2]^ Vertebroplasty is commonly used to treat osteoporotic vertebral fractures, but pulmonary embolism of cement or fat and bone marrow have been documented as serious or fatal complications after vertebroplasty.^[5]^ Spinal surgeons need to be aware of FES occurring during spinal surgery, which can cause serious complications in a very minority of patients.

To the best of our knowledge, there have been only seven cases of fatal FES occurring after posterior spinal fusion with instrumentation ^[6–11]^ (Table 1). FES can occur intraoperatively and postoperatively. Thus, patients with fluctuating blood pressure and unstable oxygenation during surgery should receive careful observation after surgery. Six of the seven cases received PS with bone cement augmentation (1 case), allograft bone augmentation (our case) and no augmentation (5 case). Embolisms occurring during intramedullary fixation of fracture ^[12]^ and cemented ^[13,14]^ and noncemented total hip arthroplasty ^[15]^ have been well studied by echocardiography. Similarly, in spine surgery, “echogenic material” by echocardiography passing through the right heart has been demonstrated in the several situations, including 1) probing of the vertebral body,^[16]^ 2) insertion of cement ^[17]^ or hemostatic agents ^[18,19]^ into the PS pilot hole and 3) placement of the PS.^[17,19]^ In addition, Kuhns et al^[19]^ provided histological evidence that hemostatic agents embolize to the lungs. Likewise, in the present case, a histological examination of lung tissue sections revealed multiple fat and small necrotic bone fragments, probably derived from the allografted bone.

**Table 1:** Cases of fatal pulmonary fat embolism after or during posterior spinal fusion surgery.

Authors	Age	Sex	Diagnosis	Operated Level	PS	PS with augmented	Onset of FES	Symptom at onset	Days from onset to death
Gittman^[7]^	17	Female	Scoliosis	T5-L4	Not used	Not used	Immediately after surgery	Shock vitals (hypotension, hypoxia)	POD 10
Brandt ^[8]^	56	Female	Spinal stenosis	L4-S1	Used	Not used	6 h after surgery	unconscious	POD 0
Temple ^[9]^	61	Female	Spinal infection	T4-L4	Used	Cement	During surgery	Shock vitals (hypotension, hypoxia)	POD 0
Joffe ^[10]^	11	Male	Neuromuscular Scoliosis	T4-L3	Used	Not used	During surgery	Shock vitals (hypotension, hypoxia)	POD 0
Stroud ^[11]^	17	Female	Neuromuscular Scoliosis	T1-sacrum	Used	Not used	24 h after surgery	sudden cardiac arrest	POD 1
Takahashi^[12]^	57	Male	Spinal infection	T1-T6	Used	Not used	5 h after surgery	suddenly dyspnea	POD 0
Our case	78	Female	Degenerative scoliosis	L2-iliac	Used	Allograft	During surgery	Shock vitals (hypotension, hypoxia)	POD 3

FES = fat embolism syndrome, POD = postoperative day, PS = pedicle screw.

To our knowledge, this is the first report to show histological evidence that the allografted bone embolized to the lungs. Therefore, although several reports have indicated that inserting reinforcing materials into the tapped screw holes (i.e. cement,^[20]^ hydroxyapatite,^[21]^ allograft bone ^[22]^) can enhance PS fixation, they might also cause severe pulmonary embolism from fat and debris of material augmentation. Spinal surgeons need to be aware of the risk of FES associated with pressurization of the vertebral body during commonly performed spinal trauma and surgical procedures.

There were several possible risk factors for FES, such as multilevel spinal surgery, impaired cardiopulmonary function, obesity, and osteoporosis.^[18,19]^

However, there is no specific treatment for FES besides ventilation management. Limiting the increase in intraosseous pressure during long bone surgery may reduce the incidence of FES.^[23]^ In spinal procedures, using the vertebral pulsed jet lavage technique to remove bone marrow from the vertebral body prior to cement injection has been reported to reduce the increased intraosseous pressure,^[24,25]^ but this remains to be verified.

FES is relatively uncommon and subclinical in most patients, and its risk factors and treatments are still unknown. Large-scale clinical studies are needed to identify patients at high risk and determine the appropriate treatments for FES.

## Acknowledgments

The authors thank Prof. Y. Sakaguchi (Department of Anesthesiology, Faculty of Medicine, Saga University) for his cooperation in suggesting skilled surgical anesthesia findings and Prof. S. Kimura and Dr. M. Yoshimura (Department of hematology, Faculty of Medicine, Saga University) for their helpful discussions.

## Author contributions

**Conceptualization:** Tadatsugu Morimoto, Takaomi Kobayashi, Masaaki Mawatari.

**Data curation:** Tomohito Yoshihara, Masatsugu Tsukamoto.

**Formal analysis:** Takaomi Kobayashi, Tomohito Yoshihara.

**Funding acquisition:** Tadatsugu Morimoto.

**Investigation:** Tadatsugu Morimoto, Takaomi Kobayashi, Keita Kai.

**Methodology:** Tadatsugu Morimoto.

**Project administration:** Tadatsugu Morimoto.

**Resources:** Tadatsugu Morimoto.

**Software:** Takaomi Kobayashi.

**Supervision:** Masaaki Mawatari.

**Validation:** Tadatsugu Morimoto, Masaaki Mawatari.

**Visualization:** Tadatsugu Morimoto.

**Writing – original draft:** Tadatsugu Morimoto.

**Writing – review & editing:** Masatsugu Tsukamoto, Masaaki Mawatari.
